# Double laparoscopy assisted cylindrical abdominal–perineal resection for low rectal cancer with 4 cases report

**DOI:** 10.1097/MD.0000000000009995

**Published:** 2018-02-23

**Authors:** Kexin Shen, Xiaofeng Cui, Zhongshi Xie

**Affiliations:** Department of Gastrointestinal Colorectal and Anal Surgery, China-Japan Union Hospital of Jilin University Changchun, Jilin, P. R. China.

**Keywords:** cylindrical abdominal–perineal resection, laparoscopy, rectal cancer

## Abstract

**Introduction:**

Rectal cancer is a common cancer worldwide. Low rectal cancer exhibits a tendency for recurrence. Surgical resection is an important treatment for rectal cancer. Cylindrical abdominal–perineal resection is suitable for patients with low rectal cancer and has helped improve the prognosis of these patients. However, there are some difficulties during the operation. Especially the perineal area operation cannot be performed under direct vision, which affects the quality of surgical resection. To resolve these constraints, our group designed double laparoscopy assisted cylindrical abdominal–perineal resection for low rectal cancer.

**Conclusion:**

The procedure effectively solved these problems and reduced the operation time with no increase in surgery complications.

## 1 Introduction

Ever since Professor Holm put forward the concept of cylindrical abdominal–perineal resection (CAPR),^[1,2]^ the prognosis of patients with low rectal cancer who undergo Miles operation has improved objectively. During the CAPR operation, the patient needs to be turned over in a folding position for perineal surgery. This has been a major constraint for the surgeons. Therefore, many scholars have tried to improve this procedure. In our experience, the difficulty of perineal operation can hardly be reduced by transabdominal abscission of levator ani in some rectal cancer patients, especially fat patients or male patients with a narrow pelvis. The perineal operation cannot be performed under direct vision, and the removal of the surgical specimen is guided by tactile sensations of the surgeon's hand; this often affects the quality of surgical resection. Using the “bottom to top” operative concept of transanal total mesorectal excision (taTME) as a reference, we performed double laparoscopy assisted cylindrical abdominal–perineal resection in 4 patients during the last 2 months of 2015.

## 2 Methods

### 2.1 Physical data

Of the 4 patients with rectal cancer, 3 patients were male and 1 patient was female. The mean age was 55.3 (47–75) years. The diagnosis of rectal cancer was confirmed by preoperative colonoscopy and pathological examination. One patient had diabetes mellitus, while the other 3 patients had no concomitant diseases. Owing to the patients’ preferences, none of the patients underwent neoadjuvant therapy. MRI examination was used for preoperative clinical staging. The results showed that 1 patient had stage II disease and the other 3 patients had stage III disease. All patients had signed informed consent before the operation.

### 2.2 Operation method

**Figure 1:**
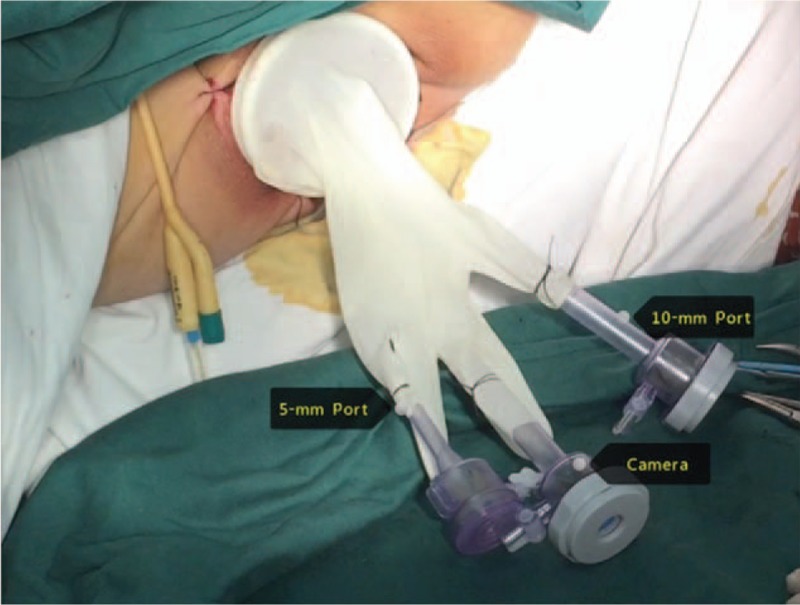
The self-made operating platform. Lap-protector was used for closing the incision. 10 mm, 5 mm, and 10 mm trocars were, respectively, placed into the thumb, little finger, and middle finger position of one rubber glove. Then the glove was set to the outside part of the lap protector.

**Figure 2:**
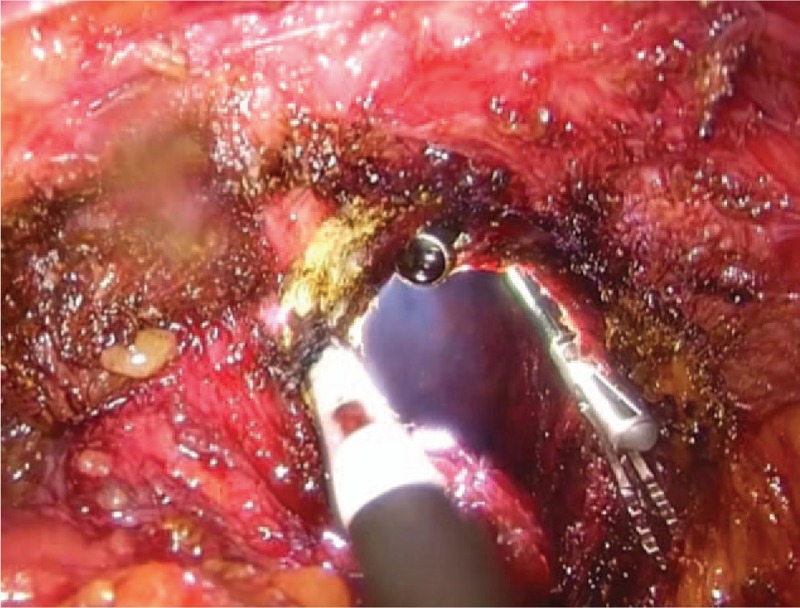
The perineal and abdominal surgery group met at the anal levator position.

The abdominal and perineal surgeries were carried out simultaneously by 2 groups of surgeons. For the abdominal surgery, we used the traditional laparoscopy assisted radical rectectomy. For the perineal surgery, the anus was closed using purse string suture. Then the skin and subcutaneous tissue were cut open at a site 3 cm away from the anus in a spindle shape from the middle of the perineum to the apex of the coccyx. The self-made operating platform was placed into the established perineum gap (Fig. 1). Lap protector was used for closing the incision. 10 mm, 5 mm, and 10 mm trocars were, respectively, placed into the thumb, little finger, and middle finger position of 1 rubber glove and then the glove was set to the outside part of the lap protector. The air pressure of the perineal surgery area was maintained between 0.9 and 1.2 kPa. The rectal specimen was pushed to the head side after ligature of the inferior mesenteric vessels. The dissection of posterolateral rectal wall proceeded along the fatty tissue layer outside of the sphincter to the anal levator initial plane under direct vision. The anal levator was then divided under guidance from both the perineal and abdominal surgery groups, and thus the 2 surgery groups could meet here (Fig. 2). During dissection of the anterior rectal wall, attention should be paid to protect urethra and prostate (vagina in female). By virtue of the magnified view afforded by the laparoscope, the muscle fibers of the superficial transverse muscle of perineum were verified as being perpendicular to the dissection plane. After severing the superficial transverse muscle of perineum, the puborectalis was exposed and after severing the puborectalis, it was possible to reach the plane of seminal vesicles (cervix in female). Thus the 2 surgery groups could easily meet and complete the dissection of tumor. Rectum and sigmoid colon were cut off at the location where the colostomy was designed using endo-GIA stapler. The specimen was taken out from the perineum. After dissecting through the extraperitoneal space, the sigmoid colon was lifted out from left lower abdomen, and a permanent extraperitoneal colostomy was carried out.

## 3 Results

The operation was successfully performed in all 4 patients, without any conversion to open surgery. The duration of operation was 280, 260, 220, and 210 minutes, respectively; the intraoperative bleeding volume was 30, 80, 120, and 20 mL, respectively. There were no serious complications. All 4 patients were able to drink water after emergence from anesthesia, and consumed some liquid diet from the second day after operation. The stoma exhaust time was 4 to 9 hours after operation. The duration of indwelling urinary catheter was 3 to 10 days. The mesorectum integrity macroscopic score of all surgical specimens was stage 3 (Nagtegaal classification). The circumferential resection margin (CRM) of the 4 specimens was negative. The TNM staging was stage III for all patients. One patient with comorbid diabetes developed incision infection in the perineal region and experienced delayed healing. Data pertaining to the 4 patients are described in Table 1.

**Table 1:**
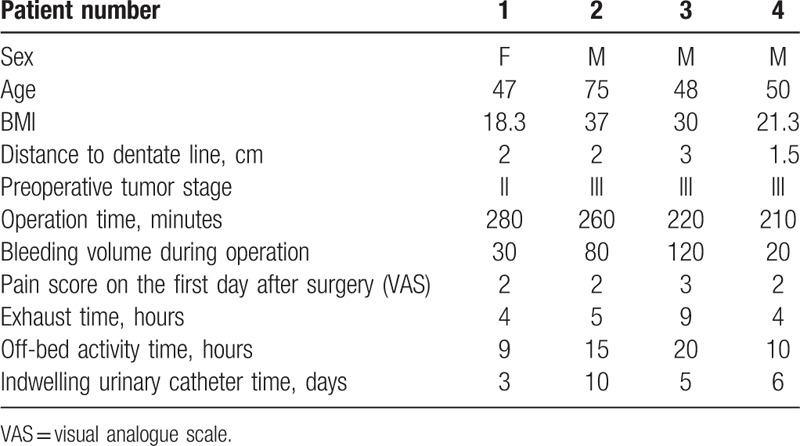
The information of 4 patients who underwent double laparoscopy assisted cylindrical abdominal perineal resection.

## 4 Discussion

During laparoscopy assisted abdominal–perineal resection, it is not possible to perform the abdominal and perineal surgery simultaneously. In order to overcome this constraint and to perform the perineal surgery under direct vision, our group designed the double laparoscopy assisted abdominal–perineal resection procedure. This procedure allows for direct visual access to the perineal operative field, which improves the precision of dissection. The conceptual design of this procedure is derived from the clinical thinking of “bottom to top” in taTME.^[3,4]^ The aim was to offer a choice when the surgeon wants to achieve the operation quality of cylindrical resection but would not like to turn the patient over to folding position during the operation. Since the perception of the various anatomical layers most often depends on the surgeon's tactile sensation, it is sometimes hard to identify the correct anatomical layer. Cylindrical resection performed in a folding position indeed provides a good view of anatomy; however, it is a challenge to achieve precision operation without turning the patient over during the operation. With our technique, the magnified view afforded by the laparoscope allows direct visual access, which helps identify the anatomical layer. Thus the boundary of the removed tissue could be clearly identified; further, the surgeon could easily achieve R0 resection. On the other hand, the magnified laparoscopic view allows the surgeon a new view angle to observe perineal topography. From the perspective of saving time, in laparoscopy assisted Miles procedure, performing the perineal operation first is liable to cause CO_2_ air leakage. Therefore, surgeons always start the perineal operation once the abdominal surgery group has divided anal levator. This certainly prolongs the operation time as well as the anesthesia time. However abdominal–perineal resection for low rectal cancer performed by laparoscopy assisted abdominal and perineal surgery groups simultaneously could preliminarily solve this problem.

Double laparoscopy assisted CAPR provides a better choice for patients with low rectal cancer and the surgeon. This procedure affords better visual access to the perineal surgery region and improves the quality of TME in CAPR. Furthermore, compared with traditional single laparoscopy assisted APR, this procedure reduces the operation time and anesthesia time; thus, it could decrease surgery related complications and facilitate postoperative recovery. Double laparoscopy assisted CAPR is an attempt to optimize surgery method with respect to the quality of surgical resection. Our experience with this method underlines its feasibility and efficacy. We believe that this procedure offers considerable leverage and is likely to find favor with surgeons.

## 5 Conclusions

Traditional operation can hardly reduce the difficulty of perineal operation, which affects the quality of surgery resection. Double laparoscopy assisted cylindrical abdominal–perineal resection could preliminarily solve this problem and reduce operation time. This procedure offers great potential for clinical application.

## References

[1] Holm T Ljung A Häggmark T Extended abdominoperineal resection with gluteus maximus flap reconstruction of the pelvic floor for rectal cancer. Brit J Surg 2007;94:232–8. 17143848 10.1002/bjs.5489

[2] West NP Finan PJ Anderin C Evidence of the oncologic superiority of cylindrical abdominoperineal excision for low rectal cance. J Clin Oncol 2008;26:3517–22. 18541901 10.1200/JCO.2007.14.5961

[3] Heald RJ A new solution for some old problems: Transanal TME. Tech Coloproctol 2013;17:257–8. 23519984 10.1007/s10151-013-0984-0

[4] Rouanet P Mourregot A Azar CC Transanal endoscopic protectomy: An innovative procedure for difficult resection of rectal tumors in men with narrow pelvis. Dis Colon Rectum 2013;56:408–15. 23478607 10.1097/DCR.0b013e3182756fa0

